# Supplementary material for: Mevalonate pathway activation in Ewing sarcoma reveals a 3D-specific synergy between statins and BCL-xL inhibition
Source: Mol Ther Oncol. 2026 May 9;34(2):201229. doi: 10.1016/j.omton.2026.201229 (PMC13251664; doi:10.1016/j.omton.2026.201229)
Supplement: Document S1. Figures S1–S5 [file mmc1.pdf]

OMTON, Volume *34*

Supplemental information

Mevalonate pathway activation in Ewing sarcoma reveals a 3D-specific synergy between statins and BCL-xL inhibition

Branka Radic-Sarikas, Marica Markovic, Martha Magdalena Zylka, Caterina Sturtzel, Mathias Ilg, Didier Surdez, Martin Metzelder, Martin Distel, Aleksandr Ovsianikov, Florian Halbritter, and Heinrich Kovar

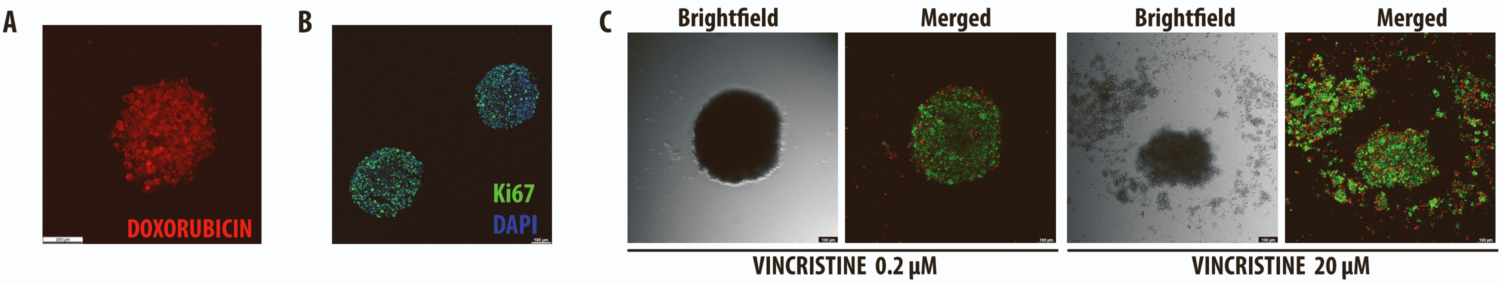

Figure S1. Visualization of proliferation, viability, and drug penetration in sarcoma models using confocal microscopy. (A) Representative central optical section (middle slice) of a confocal Z-stack of doxorubicin-treated STA-OS-5 spheroids on day 6, demonstrating drug penetration via intrinsic autofluorescence of doxorubicin. **(B)** Cryosections of OS143B spheroids at day 4, stained for DAPI (nuclei, blue) and Ki67 (proliferation marker, green). **(C)** LIVE/DEAD viability assay of vincristine-treated STA-ET-1 cells showed no effect at 0.2 µM, while 20 µM induced substantial cell disruption. Live cells fluoresced green, dead cells red.

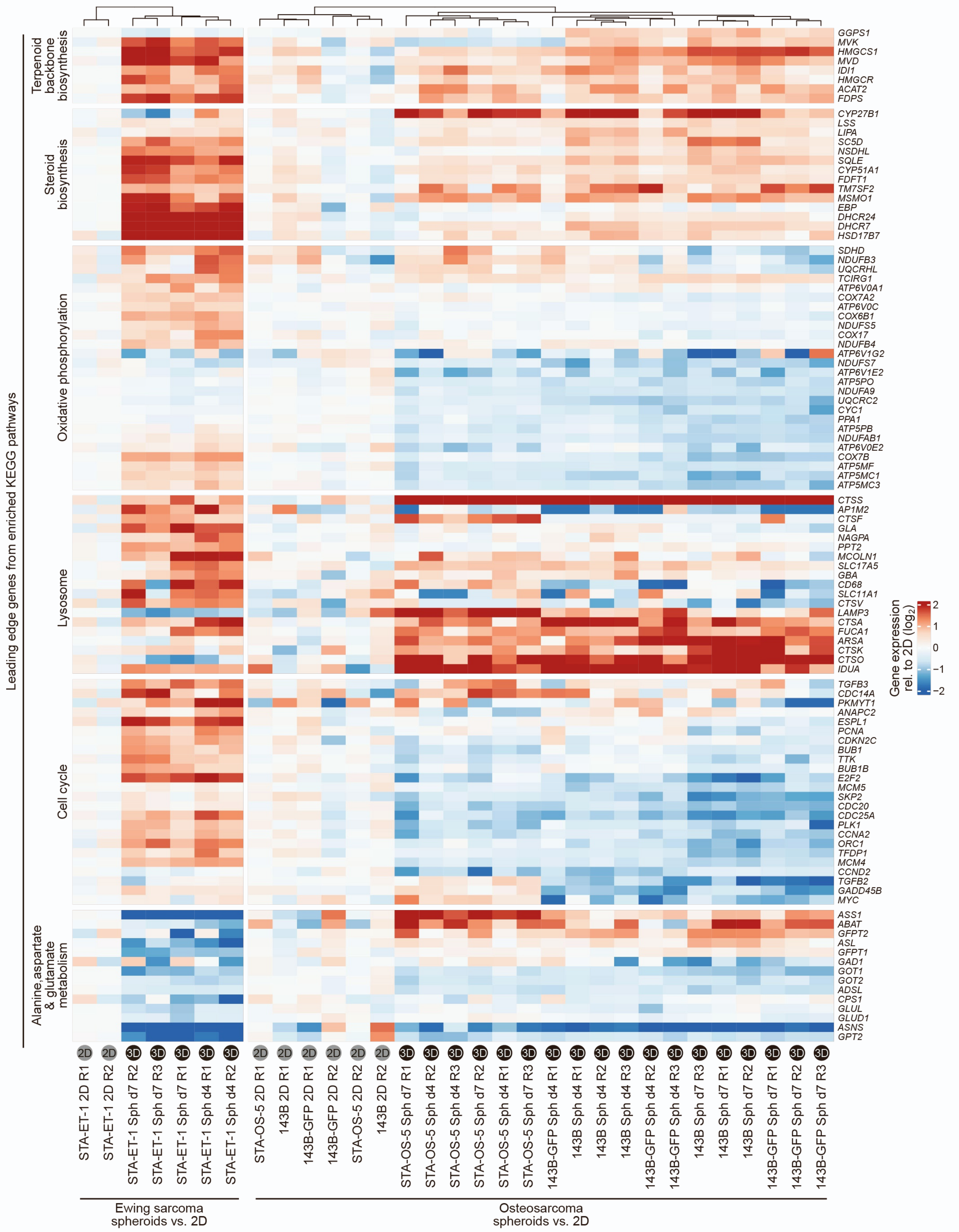

Figure S2. Pathway-level transcriptomic shifts in 3D ES and OS spheroids. Gene set enrichment analysis (GSEA) of RNA-seq data comparing 3D spheroids (d4 and d7) versus 2D cultures in Ewing sarcoma and osteosarcoma cell lines. Signiÿcant enrichment was observed for lysosomal activity, steroid biosynthesis, terpenoid backbone biosynthesis (components of the mevalonate pathway), and other metabolic and stress-response pathways. Enrichment was calculated using fgsea33 with FDR-adjusted p-values ° 0.005.

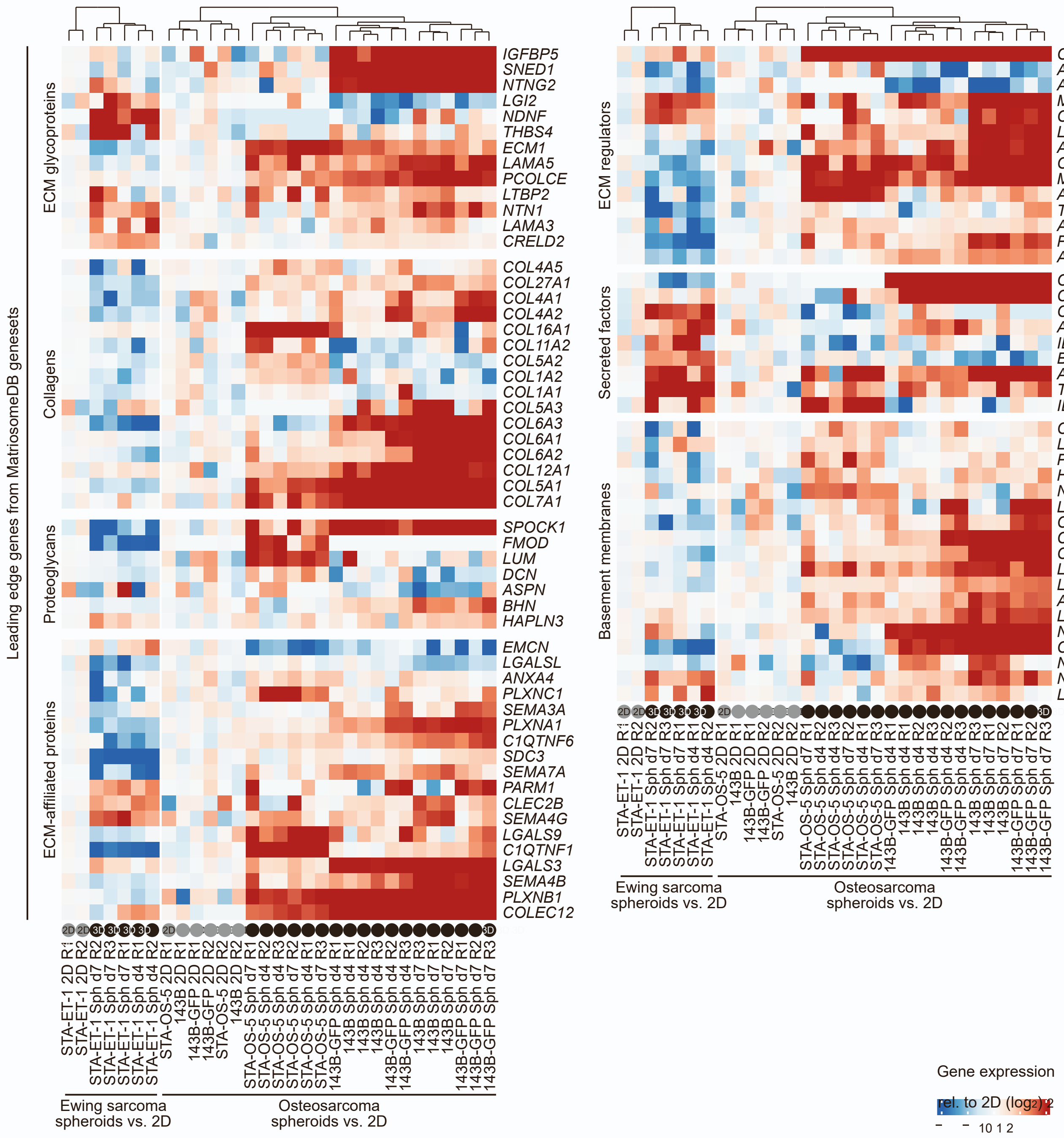

Figure S3. Differential expression of matrisome-associated genes and secreted factors in 3D cultures. Heatmap showing di˜erential expression of matrisome genes and secreted factors annotated in MatrisomeDB°˛. Osteosarcoma spheroids showed broad upregulation of genes encoding extracellular matrix (ECM) components and regulators. In contrast, Ewing sarcoma spheroids showed more selective upregulation of ECM glycoproteins and secreted factors such as IL23A, BMP6, and TNFSF12 when compared to 2D

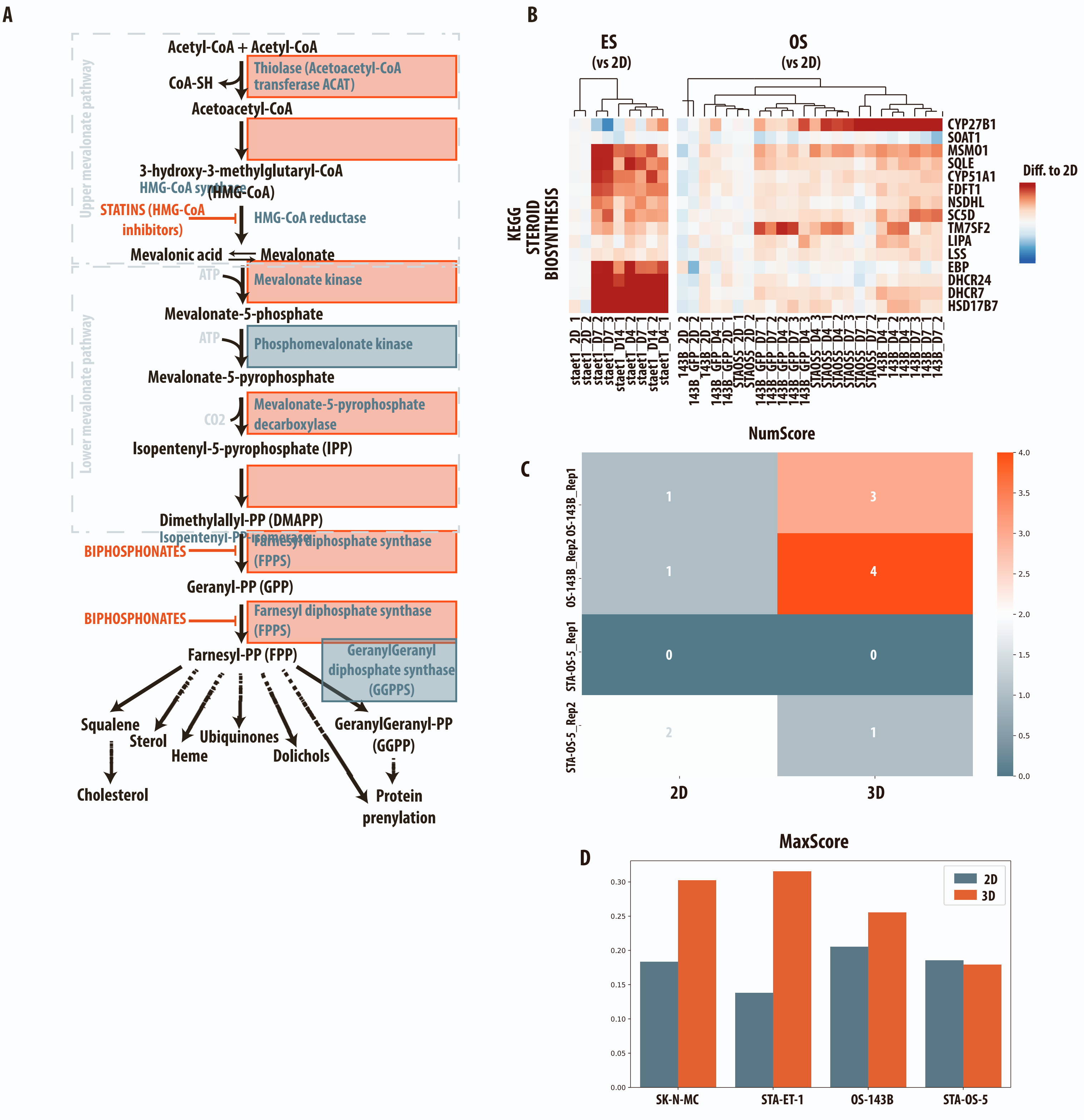

Figure S4. Differential regulation of the mevalonate pathway and drug synergy in ES and OS 3D cultures. (A) Schematic of the mevalonate pathway showing differential gene expression in ES spheroids versus 2D cultures. Upregulated enzymes are marked in red and downregulated enzymes in blue, highlighting widespread pathway activation, particularly within nodes involved in steroid biosynthesis and isoprenoid production. **(B)** Heatmaps comparing gene expression changes in the KEGG steroid biosynthesis pathway between 2D and 3D cultures for OS and ES cell lines. OS models showed limited and variable deregulation, whereas ES spheroids displayed consistent upregulation of key components, supporting enhanced pathway activity in 3D. Color scale indicates normalized enrichment score (NES), which reflects the degree of enrichment of a gene set normalized for gene set size and multiple testing. **(C)** Heatmap summarizing synergy (via NumScore) of pitavastatin and A1155463 in 2D vs. 3D osteosarcoma cultures. Compared to ES, synergy was weaker or absent in osteosarcoma, with STA-OS-5 showing a reversed effect, where activity was stronger in 2D cultures. **(D)** Comparison of MaxScore values between 3D and 2D cultures in two ES and two OS cell lines. Overall, MaxScore was higher in ES, with more pronounced 3D vs. 2D differences. STA-OS-5 exhibited a mild reversed effect, with stronger activity in 2D.

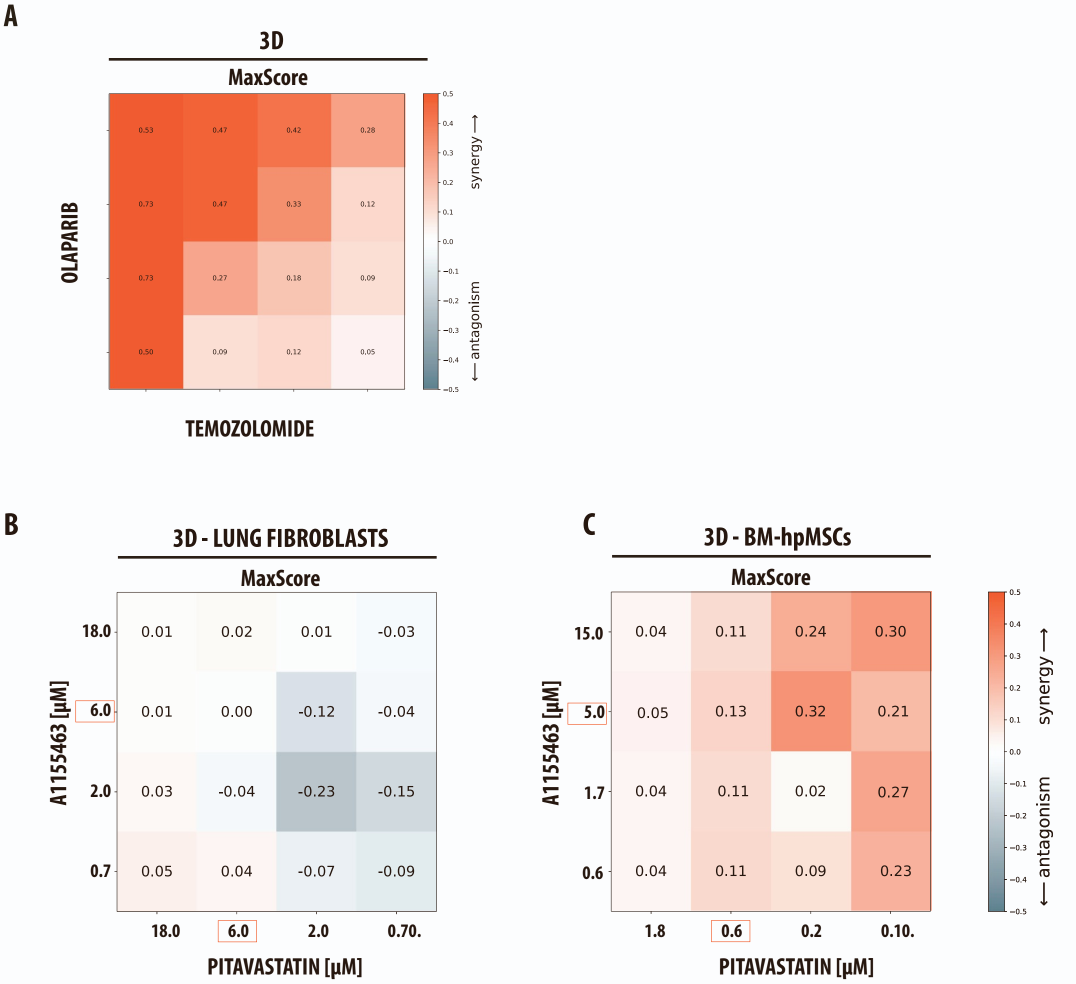

Figure S5. Temozolomide–olaparib synergy validation and evaluation of the statin–BCL xL combination in non- malignant human cells. (A) Heatmap summarizing synergy (via MaxScore) of temozolomide and olaparib in 3D Ewing sarcoma cultures (SK-N-MC cells). Despite minimal single agent activity of TMZ, the optimized dose matrix revealed a strong and reproducible synergistic interaction (MaxScore up to 0.7). **(B)** Synergy matrix for patient derived lung fibroblasts treated with pitavastatin and A 1155463. No synergistic interaction was observed; several dose pairs displayed antagonism (MaxScore down to -0.23), indicating that the combination is not broadly cytotoxic in non malignant somatic cells. **(C)** Synergy matrix for human bone marrow–derived pediatric MSCs treated with pitavastatin and A 1155463. A modest synergistic interaction
